# An explanatory analysis of economic and health inequality changes among Mexican indigenous people, 2000-2010

**DOI:** 10.1186/1475-9276-13-21

**Published:** 2014-02-27

**Authors:** Edson Servan-Mori, Pilar Torres-Pereda, Emanuel Orozco, Sandra G Sosa-Rubí

**Affiliations:** 1 National Institute of Public Health, Avenue Universidad 655, Colonia Santa María Ahuacatitlán, Cuernavaca, Morelos, Mexico

**Keywords:** Indigenous, Inequity, Development, Poverty, Health, Mexico

## Abstract

**Introduction:**

Mexico faces important problems concerning income and health inequity. Mexico’s national public agenda prioritizes remedying current inequities between its indigenous and non-indigenous population groups. This study explores the changes in social inequalities among Mexico’s indigenous and non-indigenous populations for the time period 2000 to 2010 using routinely collected poverty, welfare and health indicator data.

**Methods:**

We described changes in socioeconomic indicators (housing condition), poverty (Foster-Greer-Thorbecke and Sen-Shorrocks-Sen indexes), health indicators (childhood stunting and infant mortality) using diverse sources of nationally representative data.

**Results:**

This analysis provides consistent evidence of disparities in the Mexican indigenous population regarding both basic and crucial developmental indicators. Although developmental indicators have improved among the indigenous population, when we compare indigenous and non-indigenous people, the gap in socio-economic and developmental indicators persists.

**Conclusions:**

Despite a decade of efforts to promote public programs, poverty persists and is a particular burden for indigenous populations within Mexican society. In light of the results, it would be advisable to review public policy and to specifically target future policy to the needs of the indigenous population.

## Introduction

It is well known worldwide that indigenous people are economically and socially deprived when compared to non-indigenous populations [1]. Indigenous people are worse off in terms of health for most indicators [2,3]. In Latin America, these populations have historically been among the poorest and most excluded subpopulations at the country-level [3,4]. They have not only faced serious disadvantages in terms of their basic rights to their ancestral property, languages, cultures, and forms of governance, but also in terms of inequitable access to basic social services such as health services, education, and infrastructure, among others.

Overcoming this social and health inequity gap in indigenous people is a recognized high priority in many countries and is often included in the national public agenda. An international commitment was further demonstrated in the 2006 signing of the United Nations Declaration (UN) on the Rights of Indigenous Peoples, which recognizes that “Indigenous peoples have the right, without discrimination to the improvement of their economic and social conditions, including, inter alia, in the areas of education, employment, vocational training and retraining, housing, sanitation, health and social security” [5].

Mexico with a population of 112,336,538 people (according to the last population census 2010) [6] is an upper-middle-income country [7] and the second largest economy in Latin America [8], with a constitutional federal democracy ruled by a president. The distribution of the country’s wealth is inequitable, with a large divide between the resources of the rich and poor. According to the UN’s Human Development 2013 report “The Rise of the South”, Mexico is ranked 61st in development worldwide, with a score of 0.775 on the human development index [9]. There are 67 indigenous languages spoken, and at least 65 indigenous ethnic groups distinguished from each other on the basis of linguistic criteria [10].

Socio-economic inequality characterizes ethnic groups in Mexico. In 2010, there were 6.7 million individuals aged ≥5 (6.8% of the total population) [6] who spoke at least one indigenous language, were characterized as structurally vulnerable and were living in poor conditions [11]. Indigenous communities live in environments with higher levels of poverty, have worse health outcomes, lower life expectancies, and poor academic performance – all causal and consequential elements of poverty [1].

Sensitive maternal and child health indicators highlight the disadvantage that indigenous people experience. For example, maternal mortality among indigenous women is as much as five times higher than that among non-indigenous women [12], and one out of four indigenous women has no access to family planning methods [13]. Additionally, at least 60 per cent of indigenous women who were pregnant had iron deficiency at the time of delivery [14]. Lastly, the prevalence of child malnutrition among indigenous children was 44 per cent in comparison to 17 per cent among non-indigenous children [15], and the infant mortality rate was 50 per cent higher among indigenous children compared to non-indigenous children [15,16].

In Mexico, despite international treaties meant to ensure the well-being of indigenous populations, such as the Agreement No. 169 signed in 1989 with the International Labor Organization (ILO) [17] and the recently adopted resolution of health and human rights that prioritizes indigenous peoples [18], there are very few initiatives that target improving indigenous wellbeing. The initiatives that do exist are scattered, approaching a wide variety of topics including cultural, legislative, and poverty-related challenges without consideration of the national social policies that have been instituted to reduce poverty in Mexico in a rigorously monitored system. Thus, the effects of the initiatives for indigenous peoples often have unknown outcomes since they are not accompanied by evaluation components.

In the last fifteen years, the human development program Oportunidades [19] has been the most important ground-breaking initiative at the international level of public policy oriented at directly fighting poverty in Mexico. This program has been conceived as a transitional social support program targeted at the household level. It grants seven of the ten percent national GDP allocated to social spending in order to carry out investments in human capital through conditioned cash transfer mechanisms. The program encourages the use of public health, education, and nutrition services and is focused on breaking the intergenerational cycle of poverty [20].

Although Oportunidades has shown positive effects on basic health, nutrition, and education indicators among disadvantaged groups [19,21-25], some marginalized indigenous communities have not been included in the program because of the localities where they live. These areas do not meet the minimum requirements for the program’s inclusion. These communities are extremely marginalized and that do not have a school or a rural health center that would allow them to meet inclusion criteria. The existing literature on the effects of Oportunidades on the well-being of the Mexican indigenous population, suggests major problems about program adequacy and challenges in achieving good participation rates [26]. The challenges have been attributed to cultural and structural barriers, differential access to health services, the need for the program to be modified to the traditions and the way of life of indigenous people [27], and the assessment of lack of correspondence between the years of education attained by women and their persistent rates of low-income employment [26,28].

Despite the importance of studying indigenous populations in the context of developmental strategies, there is little quantitative research that documents ethnic inequalities in Latin American development, especially in Mexico [2]. This study examines the social disparities according to ethnic condition in terms of changes in socioeconomic status, poverty levels, and health (childhood stunting^a^ and infant mortality) for the time period 2000–2010.

## Methodology

### Population definition and data

According to the Mexican National Commission of Indigenous Peoples Development, an indigenous person is defined as someone “living in a household whose head of the family, a spouse and/or an ascendant self-identifies and speaker of an indigenous language” [29].

Initially, we explored the correlation between indigenous presence at the municipality level, and different economic development outcomes, such as housing conditions (defined as section of household without durable flooring material and without access to clean water and sanitation), medical insurance, illiteracy, food poverty [30], and human development index [31].

Secondly, we investigated the economic and health inequalities, analyzing the distribution of household expenditure (as proxy of income, in deciles) comparing indigenous and non-indigenous population groups in two periods of time (2002 and 2010). We also investigated modifications in the levels of poverty, using poverty indexes and comparing the same groups in 2002 and 2010. Finally, we explored health conditions of the two most relevant child health outcomes [32-34]: childhood stunting and infant mortality. Data for this study was extracted from five statistical sources:

i. The Mexican Census 2005 [35] and data coming from the United Nations Program for Development from which the proportion of indigenous people at municipality level was extracted [31].

ii. The Mexican National Household Survey of Income and Expenditures (NHIES) 2010 [36]. This is a representative (national and rural/urban strata), and a cross-sectional survey of households, undertaken by the National Institute for Statistics, Geography, and Informatics. Detailed and standardized questions are asked about all sources of income and categories of expenditure (including a module on health at household level). The survey also includes family style, labor market status, and education. The time frame, methods, and questionnaires are consistent and comparable across years.

iii. The Mexican Family Life Survey (MxFLS) 2002. This is the first Mexican survey with national representation taken from a longitudinal design. It is a multi-thematic and longitudinal database which collects, with a single scientific tool, a wide range of information on socio-economic, demographic and health indicators of Mexican population [37].

iv. The Mexican Survey of Health and Nutrition (NHNS, 1988–2012) [38,39]. It has been conducted since 1988 each five years, making it possible to determine the nutritional status of Mexican population based on national probability surveys of rural and urban strata in different regions of the country; and

v. We complemented these datasets with original data on infant mortality applied in the most recent Human Development Report on Indigenous Peoples in Mexico, 2000, 2005 and 2010 [40].

### Analytical strategy

Our first step was to describe the economic profile of indigenous people at the municipality level, by combining locality level information from the 2005 Mexican Census and UNDP. This data concerns the availability of basic housing conditions (as defined previously); percentage of illiteracy; low food poverty and the proportion of people without medical insurance. The human development index was used as an additional indicator. This index is a composite statistic of life expectancy, education, and income indices used to rank municipalities into human development [41].

Secondly, an inequality analysis of ethnic condition was performed using MxFLS 2002 and NHIES 2010 with two sets of different indicators: (a) the change in the distribution of the household equivalent expenditure^b^ (in deciles) at 2010 prices [42], and (b) the change in the levels of poverty between 2002 and 2010 across indigenous and non-indigenous populations by means of an estimation of two of the most important poverty indexes: the Foster-Greer-Thorbecke (FGT) [43], and Sen-Shorrocks-Sen (SST) [44]. The FGT index is based on the estimation of a standardized gap between a poor person and a wealthy individual, which is the income shortfall expressed as a proportion of the poverty line. The SST index combines measurements of the proportion of poor people, the depth of their poverty, and the distribution of welfare among the poor. These estimations were performed using different poverty line thresholds (US$50, US$75, US$100).

Finally, we used public results from the Mexican National Survey of Health and Nutrition (1988 and 2012) and the last Human Development Report on Indigenous Peoples in Mexico to analyze changes in two health indicators: the prevalence of stunting in children (1988–2012) [38,39] and infant mortality rates (2000–2010) [31].

## Results

**Figure 1:**
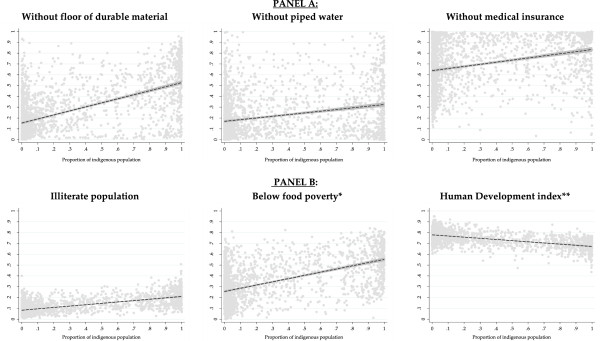
**Economic profile of the indigenous people in México 2005.** PANEL **A**: Access to public services. PANEL **B**: Indicators of level of development. Note: Estimated base on The Mexican Census 2005 [35] and data come from United Nations Program for Development – Mexico at municipality level [31]. *Refers to people who are unable to meet their basic food needs. ** A composite statistic of life expectancy, education, and income indices used to rank municipalities into human development [30].

Figure 1 describes the economic profile of indigenous people in Mexico in 2005 at the municipality level. This figure contains six graphs that show indicators of the proportion of population with access to different public services and variables that indicate the level of development at the municipality level. The vertical axes for the first three graphs show the proportion of the indigenous population living in households without durable flooring material, clean water and sanitation, and the proportion of population without health insurance respectively. The vertical axes for the following three graphs show the proportion of illiterate population, the proportion living under the food poverty line and the level of development based on the human development index. These six indicators are correlated with the proportion of the indigenous population at the municipality level. Hence, for all six graphs the horizontal axis indicates the proportion of the indigenous population at the municipality level. Overall, those municipalities with a higher proportion of indigenous population have less availability of basic services such as durable flooring material, access to clean water and sanitation and medical insurance. (Note the negative slope of graphs 1, 2 and 3). Similarly, having a higher proportion of indigenous population is associated with higher prevalence of poverty and illiteracy.

**Figure 2:**
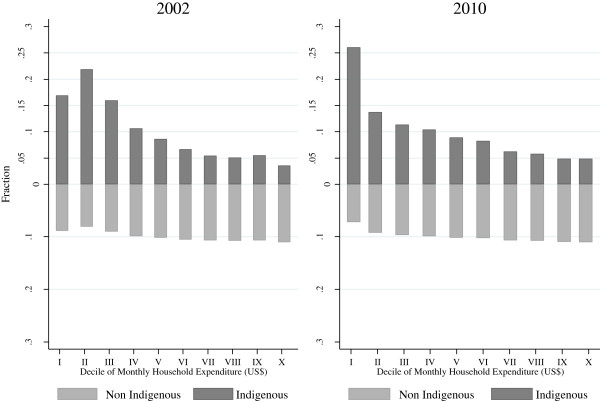
**Distribution of household expenditure by ethnic group in Mexico, 2002-2010 **[37,45]**.** Note: Data used came from The Household Survey of Income and Expenditures (NHIES) 2010, Mexican family Life Survey (MxFLS) 2002 [45,36]. The Monthly Household Expenditure was reported by Equivalent Adult at 2010 prices.

Figure 2 shows the distribution of population by deciles of total household expenditure and ethnicity in 2002 and 2010. For both years, about 50 percent of indigenous population can be found in the first three deciles of household expenditure. Additionally, compared to 2002, the proportion of indigenous population located in the first decile of household expenditure increased considerably in 2010.The distribution of household’s expenditure showed that the indigenous populations are disadvantaged economically: In 2002 about 15% of the indigenous population belonged to the first decile of household expenditure, this proportion increasing to 25% in 2010. This shows that the proportion of indigenous population with less economic capacity increased between 2002 and 2010.

**Figure 3:**
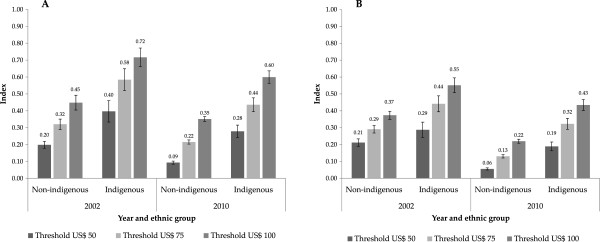
**Poverty indexes by ethnic group in Mexico.** Graph **A**. Foster –Greer-Thorbecke (FGT) index. Graph **B**. Sen-Shorrocks-Thon (SST) index [37,45]. Note: Data used came from The Household Survey of Income and Expenditures (NHIES) 2010, Mexican family Life Survey (MxFLS) 2002 [45,36]. The Poverty Lines were calculated from the Monthly Household Expenditure by Equivalent Adult at 2010 prices. 95% - Confidence intervals adjusted by ethnic group and survey design.

Figure 3 shows the FGT, and SST poverty indices estimated for 2002 and 2010 for both indigenous and non-indigenous population groups with different poverty line thresholds (US$50, US$75, US$100). Overall this figure shows that regardless of the type of poverty index estimated and the different thresholds of the poverty line, indigenous population consistently have greater poverty index values compared to non-indigenous populations.

This figure shows that the value of the indices estimated and the difference between both population groups varies, depending on the index estimated (FGT or SST indexes) and the poverty line used (US$50, US$75, US$100) however general trend remains constant. Although the poverty levels declined for indigenous and non- indigenous groups of population in the period 2002–2010, the gap between the two groups persists. Specifically, the FGT index suggests that differences between indigenous and non-indigenous groups are maintained between 20 and 25 percent in both years (see Panel A, Figure 3); or in the SST index, the differences varied between 8 and 18 percent and between 13 to 21 percent (see Panel B, Figure 3). We can observe in Figure 3 a reduction in the poverty index for both indigenous and non-indigenous populations, but there is a significant reduction among non-indigenous groups in all of the different poverty line thresholds.

**Figure 4:**
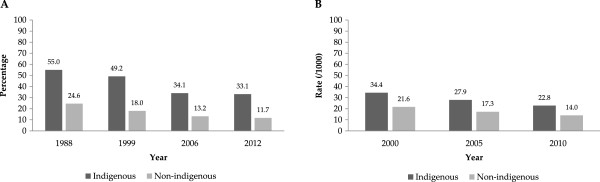
**Prevalence of childhood stunting and infant mortality rate in Mexican children, by ethnic group.** Graph **A**. Childhood Stunting in Mexican Children (< 5 yrs) [39]^*^. Graph **B**. Infant Mortality Rate [31]^**^[29,46]. * Children who are below minus two standard deviations from median height for age of reference population are classified as stunted. (Height for age <–2 SD of the WHO Child Growth Standards median) [50]. ** Probability of dying between birth and exactly one year of age expressed per 1,000 live births [46].

Finally, Figure 4 shows two health outputs sensitive to the poverty condition: the prevalence of stunting in children from 1988 to 2012 (Panel A) and rate of infant mortality from 2000 to 2010 by ethnic status (Panel B). This figure shows that in general, the prevalence of stunting in children and the rate of infant mortality have declined over time; however, there are still significant differences in the trend of these health indicators when we compare indigenous and non-indigenous groups of population. While the reduction in the percentage of childhood stunting was 42% among indigenous population groups during the period 1988–2012, this reduction was even greater (52%) among children living in non-indigenous municipalities during the same period. The same trend is evident in the infant mortality indicator. Although we can see reductions in the infant mortality rate per 1000 inhabitants in both indigenous and non-indigenous groups during 2000–2010, the average rate of decline was greater among non-indigenous groups (35.2%) compared to the indigenous populations (33.7%). Despite these important achievements in reducing infant mortality, these results reveal that indigenous people remain in an unfavorable and vulnerable position compared to their counterparts in the non-indigenous groups.

## Discussion

This analysis provides evidence of the persistent disparities between Mexico’s indigenous and non-indigenous population regarding access to basic and crucial development indicators. Mexico’s indigenous populations live in unfavorable and very vulnerable environments. Our findings indicate that efforts made over the past 10 years targeting indigenous people apparently have not been sufficient. Indigenous groups continue having limited access to health services, quality education, and employment opportunities [5,13,47]. Together these factors lead to substantial costs in terms of social welfare, which are then inherited from one generation to another [48].

Our findings suggest that although health indicators have improved over the years among indigenous population groups; health inequalities persist between indigenous and non-indigenous population groups. These results imply an accumulation of health disadvantages [26] that contribute to deepening structural inequality [11].

When we compared indigenous and non-indigenous groups, we found that although improved socio-economic and health conditions have been achieved among indigenous groups of population in the last ten years, the gaps in economic, well-being and health outcomes persist. These differences contribute to an unequal distribution of resources and opportunities, benefiting some groups of the population over others. These inequalities also limit the degree of development possible for more disadvantage population groups, reducing their ability to overcome poverty [3].

Our analysis showed positive effects on reducing poverty when indigenous and non-indigenous population groups were compared. This reduction could be due to social programs targeting the poorest groups of the population. However, these efforts apparently have not been sufficient. Although better socio-economic and health conditions have been achieved among indigenous populations, they continue facing unequal development and living conditions. Further analysis is required to define the specific strategies to reduce the gaps still present.

There are some limitations to this study. The definition of indigenous peoples for the empirical analysis was adjusted according to the specific questions available in some of the surveys. Meaning, the question of self-report inquiry about self-determination of the indigenous based on language spoken could be biased. This could obscure the actual magnitude of the differences between the indigenous and non-indigenous populations. Therefore, clearly defined indicators are needed in the future to produce more precise results. This analysis focused on specific socio-economic indicators and welfare that do not fully describe the conditions of life and the disadvantages of indigenous peoples. Finally, the authors did not attempt to undertake a causal analysis but completed an empirical analysis focused on the description of the changes in the leading indicators over time and the comparison of the trends in these indicators among population groups. Future studies should analyze the specific characteristics and contributions of social programs that have been shown to improve the socio-economic states for indigenous population groups so findings can contribute to further reducing the gap between indigenous and non-indigenous populations.

Our study has shown that population groups living in impoverished conditions do not always have the same characteristics and that indigenous population groups are likely to experience much greater disadvantage than impoverished non-indigenous groups. The 169 Agreement signed in 1989 clearly states the need to consider specific characteristics of indigenous population groups (indigenous institutions, property, work, cultures and environment) better respond to their vulnerability [49]. Therefore, it is important that programs are planned and implemented that meet the specific needs and characteristics of disadvantaged population groups such as indigenous populations.

Today, some public policies in Mexico are homogenous while seeking to address heterogeneous realities. An example of this is the above mentioned case of communities that despite their great need, given their unfavorable structural poverty conditions, are not eligible to receive the Oportunidades program because their community does not have a school or rural health center. It is crucial to refine and even redirect public interventions and efforts that for several years have approached poverty and inequality issues in Mexico through broad approaches which do not consider the specific needs of all disadvantaged sub-populations in the country.

## Endnotes

^a^Children who are below minus two standard deviations from median height for age of reference population are clasified as stunted. (Height for age < −2 SD of the WHO Child Growth Standards median) [50].

^b^The main use of equivalence scales is to provide a metric from which to perform comparisons of welfare indicators between households of different demographic composition. For the measurement of poverty, these scales are especially convenient because they take into account in their calculations, both the size and the composition of households rather than simply using total or per capita resources in comparisons against established poverty lines [51].

## Competing interests

We declare having non- competing interests.

## Authors’ contributions

ES-M has made substantial contributions to conception and design of the article; he had a central role in the acquisition of data as well as in the analysis and interpretation of data. ES-M was involved deeply in drafting the manuscript and answering the referee’s suggestions. ES-M gave his final approval of the version to be submitted for publication. PT-P has made substantial contributions to conception and design of the article; she had a central role in the analysis and interpretation of data. PT-P had a central role in the theoretical analysis of the data. PT-P was involved in drafting the manuscript and answering the referee’s suggestions. PT-P gave her final approval of the version to be submitted for publication. EO contributed in the analysis of the data. He contributed as well in continuous revisions of the drafted article. He contributed to the critical analysis of the argument and gave his final approval of the version to be submitted for publication. SGS-R made substantial contributions to the analysis and interpretation of data; she has been involved in drafting the manuscript or revising it critically for important intellectual content; and has given her final approval of the version to be submitted for publication. Each author has participated sufficiently in the work to take public responsibility for appropriate portions of the content.
